# Duration of SARS-CoV-2 viral RNA in asymptomatic carriers

**DOI:** 10.1186/s13054-020-02952-0

**Published:** 2020-05-24

**Authors:** Xiquan Yan, Xiaotong Han, Yong Fan, Zhixiong Fang, Da Long, Yimin Zhu

**Affiliations:** 1 Hunan Provincial Institute of Emergency Medicine, Hunan Provincial People’s Hospital/The First Affiliated Hospital, Hunan Normal University, Changsha, Hunan China; 2 School of Life Sciences, Hunan Normal University, Changsha, Hunan China; 3 Xiangtan Central Hospital, Xiangtan, Hunan China; 4 Department of Infectious Diseases, Loudi Central Hospital, Loudi, Hunan China; 5 Public Health Centre, Xiangtan Central Hospital, Xiangtan, Hunan China; 6 Department of Science and Education, Shaoyang Central Hospital, Shaoyang, Hunan China

**Keywords:** SARS-CoV-2, COVID-19, Asymptomatic carriers, Viral RNA, qRT-PCR

Coronavirus disease 2019 (COVID-19) is an emerging infectious disease that was first reported in Wuhan, China, and which has subsequently spread worldwide [1]. Zhang and colleagues reported in *Critical Care*, providing evidence that asymptomatic carriers can transmit COVID-19 [2]. To our knowledge, no study comprehensively investigated the duration of severe acute respiratory syndrome coronavirus 2 (SARS-CoV-2) viral RNA in asymptomatic carriers.

We present SARS-CoV-2 qRT-PCR results of all respiratory samples from 24 asymptomatic carriers with COVID-19 at the Loudi Central Hospital, Shaoyang Central Hospital, and Xiangtan Central Hospital in Hunan province, China. Respiratory samples were collected every 1–2 days until two sequential negative results were obtained. We obtained epidemiological and demographic data, as well as clinical features. The study has been approved by the ethics commission of each participating hospital. Written informed consent was obtained from the patients.

From January 29 to February 24, 2020, we enrolled 24 asymptomatic carriers. Respiratory samples were collected from all of them. The mean SARS-CoV-2 RNA carriage period, defined as the interval from the day of exposure to the first day of continuous negative tests, was 22.0 days (SD 7.1). The mean positive RNA test period, defined as the interval from the first day of positive nucleic acid tests to the first day of continuous negative tests, was 7.9 days (SD 3.5). The full disease course of the 24 patients with respiratory samples that were positive for SARS-CoV-2 RNA is shown in Fig. 1. Notably, patient 2 carried SARS-CoV-2 viral for 32 days continuously after exposure to COVID-19 and tested positive for viral RNA in the respiratory sample for 13 days after first positive test onset.

**Fig. 1:**
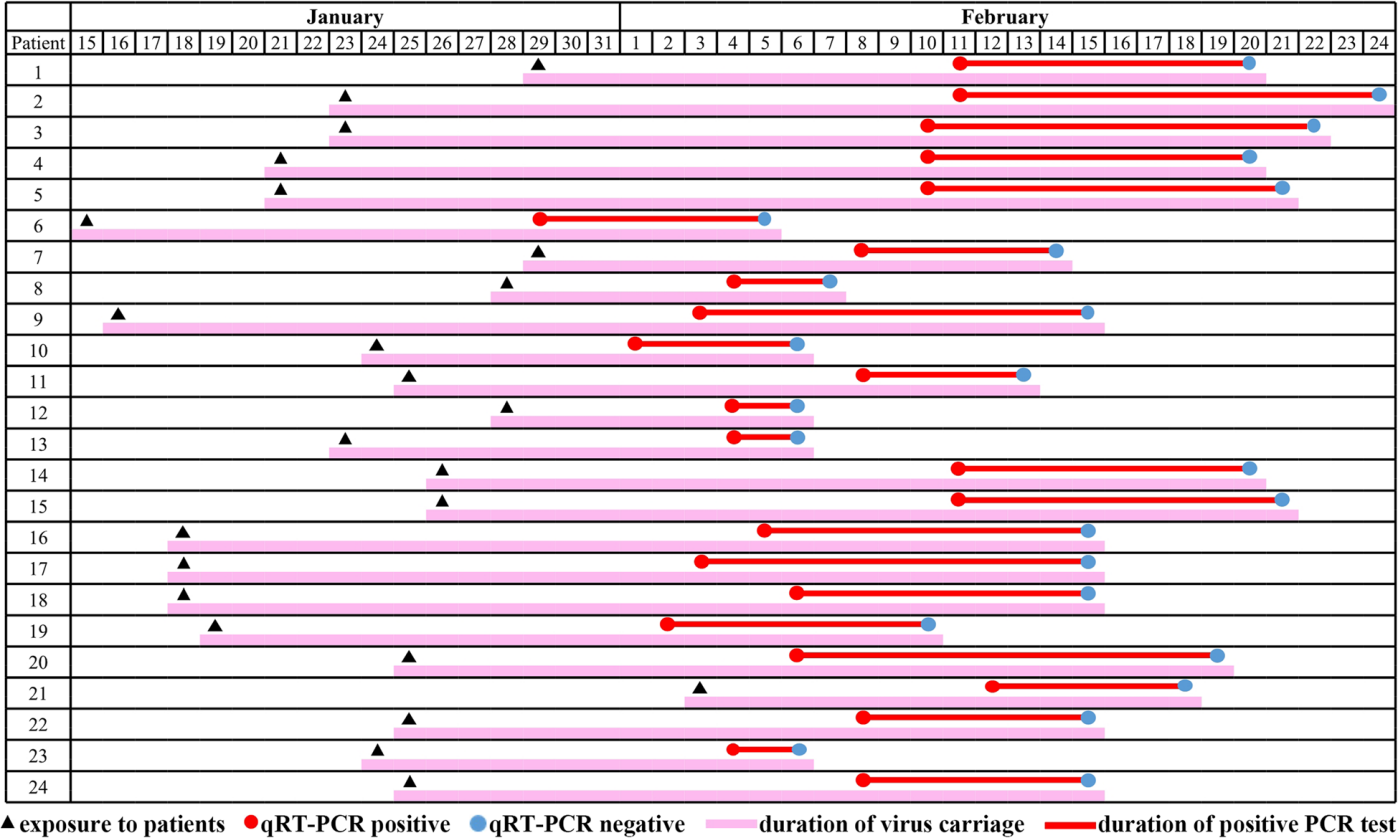
Timeline of the disease course for 24 asymptomatic carriers

The results indicate that asymptomatic human can carry SARS-CoV-2 viral RNA after exposure to COVID-19, and the carriage seems long-lived. Yet, the viability of SARS-CoV-2 detected on qRT-PCR in carriers remains to be proved by means of viral culture. Due to limited resources, we did not perform these tests. Further study is needed to determine the potential for and mode of contagion of asymptomatic carriers to develop more scientific control strategies.

The long duration of asymptomatic infection with SARS-CoV-2 may warrant a reassessment of quarantine as the current outbreak. And it is of great public health significance to strictly monitor close contacts via multiple nucleic acid screenings to contain potential outbreaks.

Zhang and colleagues provided evidence of asymptomatic transmission [2], and the relatively high proportion of asymptomatic infections could have public health implications [3]. To prevent and control this highly infectious disease in early phases, people with close contact with SARS-CoV-2 infection should be closely monitored and examined to rule out infection, even if they do not have any symptoms. The US Centers for Disease Control and Prevention recommends that contacts of asymptomatic carriers self-isolate for 14 days [4]. Social distancing is one of the most important ways to cut off transmission routes—people cannot pass on infection if they do not come into contact with other people. Quarantine of asymptomatic carriers and identification of contacts are a crucial part of these control efforts.

This study is limited by the small sample size. Large-scale multicenter studies are needed to verify our findings. There is a great need for further studies on the mechanism by which asymptomatic carriers could acquire and carry SARS-CoV-2 that causes COVID-19. These results highlighted the importance of quarantine. Hence, with extensive efforts on close contact tracing and longitudinally surveillance via SARS-CoV-2 viral RNA tests, the prevention of SARS-CoV-2 infection would prove challenging.

## Data Availability

Not applicable.
